# Carimas: An Extensive Medical Imaging Data Processing Tool for Research

**DOI:** 10.1007/s10278-023-00812-1

**Published:** 2023-04-27

**Authors:** Oona Rainio, Chunlei Han, Jarmo Teuho, Sergey V. Nesterov, Vesa Oikonen, Sauli Piirola, Timo Laitinen, Marko Tättäläinen, Juhani Knuuti, Riku Klén

**Affiliations:** Turku PET Centre, University of Turku and Turku University Hospital, Turku, Finland

**Keywords:** Image analysis, Image processing, Medical imaging, Positron emission tomography, Software

## Abstract

Carimas is a multi-purpose medical imaging data processing tool, which can be used to visualize, analyze, and model different medical images in research. Originally, it was developed only for positron emission tomography data in 2009, but the use of this software has extended to many other tomography imaging modalities, such as computed tomography and magnetic resonance imaging. Carimas is especially well-suited for analysis of three- and four-dimensional image data and creating polar maps in modeling of cardiac perfusion. This article explores various parts of Carimas, including its key features, program structure, and application possibilities.

**Supplementary Information:**

The online version contains supplementary material available at 10.1007/s10278-023-00812-1.

## Introduction

Analysis of medical images is crucial for finding the correct diagnosis of a patient, monitoring their condition, and obtaining information needed to prepare a surgery or other treatment. Several different modalities, such as positron emission tomography (PET), computed tomography (CT), and magnetic resonance imaging (MRI), are used to scan either some specific organs or larger regions of the human body. Since this type of imaging results in a three-dimensional image consisting of numerous transaxial image slices on top of each other and sometimes having also time as a fourth dimension, it is necessary to have suitable software for viewing and analyzing these images efficiently.

For this purpose, the development of Carimas began in Turku PET Centre in Turku, Finland, in 2005. The first version of the software was coded in IDL programming language in 2009 and because this version was mainly for processing $^{15}$O-labeled water myocardial perfusion imaging data [1], it was named Carimas as an abbreviation of CARdiac IMage Analysis System. Based on the success of the IDL version of Carimas, the project was developed further. The second version of Carimas was also re-written in C# with Visual Studio in 2009 as it was more convenient for professional software development. Here, we use the name Carimas to refer to this C# version of the software.

During the last decade, Carimas has become very popular. In Turku PET Centre, it is a routine imaging data processing tool for most researchers. It has currently 900 registered users around the world. So far, over 150 scientific research papers have been published using Carimas as an imaging processing tool. Research carried out with Carimas is mostly related to oncology, cardiology, and neurology.

In this article, we introduce Carimas. First, in “Key Features”, we list the key features of this software that have been used as the main principles while developing it in the first place. Then we briefly explain the technical structure of Carimas in “Technical Details”, and give more detailed examples of how Carimas can be applied in “Examples of Application”. Finally, we discuss the future of Carimas and compare it with other existing tools in “Discussion”.

## Key Features

### Complete Tool for Visualization and Analysis

**Fig. 1:**
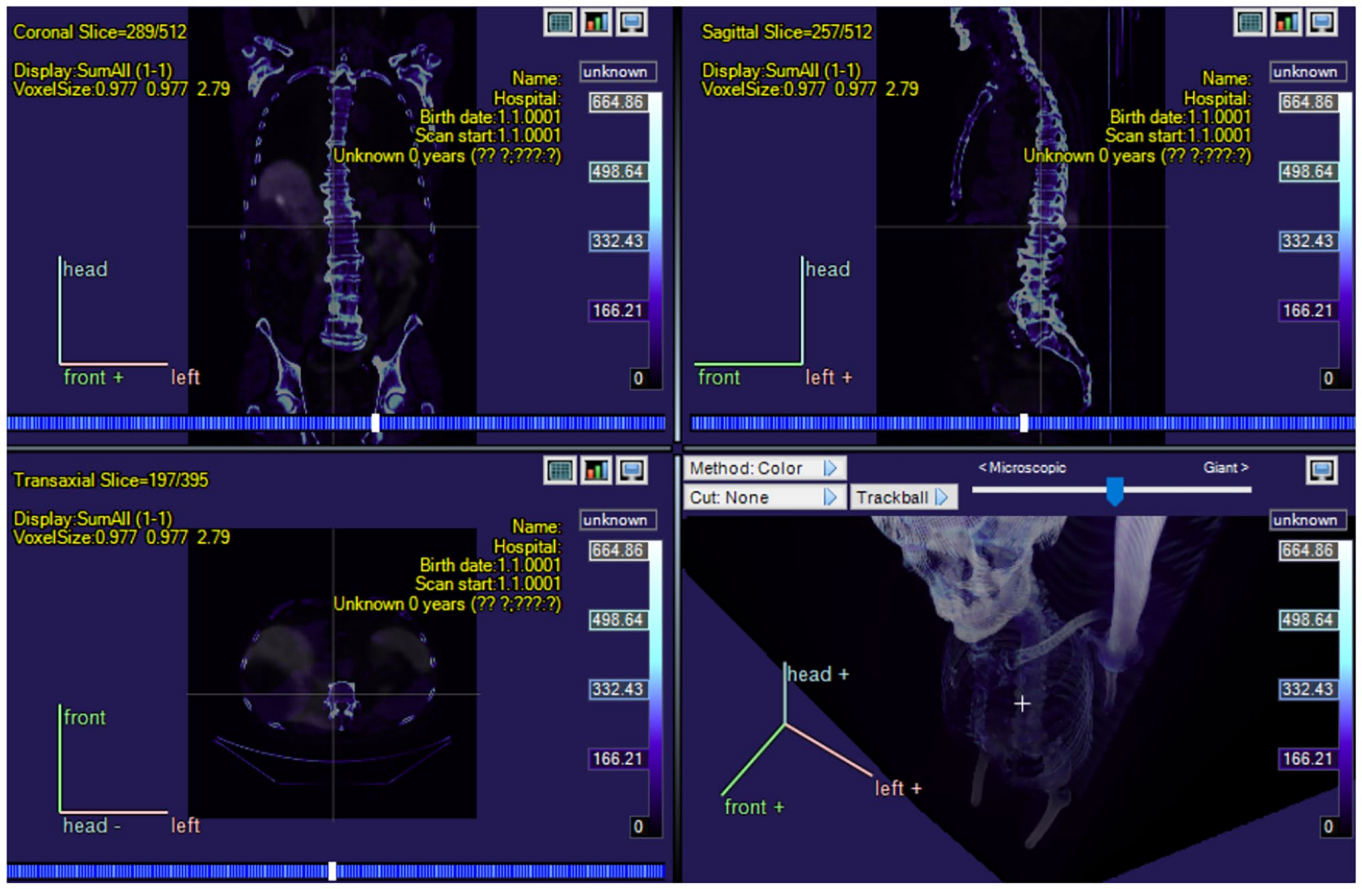
A screenshot of a whole-body PET/CT image of a prostate cancer patient opened in Carimas with color function called Ice, showing the coronal, the sagittal, and the transaxial directions and image modality fusion

For sake of efficiency, it is a considerable benefit if researchers can use a single software tool for their whole work instead of needing a different program for each subtask. This is why Carimas was designed to offer a great variety of functions related to visualization and analysis of medical images. The common workflow contains four steps: loading images, segmentation, analysis, and reporting. To visualize three-dimensional images, they can be viewed from transaxial, coronal, sagittal, or any other direction, moved and rotated freely in the three-dimensional space by the user, and divided into two-dimensional slices perpendicularly to the three aforementioned anatomic directions, as can be seen from Fig. 1. The contrast and the color pallet can be adjusted in a simple way and grayscale images can be converted into colors with multiple different functions. A two-dimensional region of interest (ROI) or a three-dimensional volume of interest (VOI) can be specified by either by drawing it directly onto the image or using semi-automatic or automatic methods, and its pixel or voxel values can be analyzed by computing their mean value, standard deviation, range, or other statistics. Furthermore, all the processing statuses and analysis results can be saved in a project file so that the work performed earlier can be restored fully in the future.

### Image Fusion and Co-Registration

Since Carimas was originally developed to be used mostly for PET images, which need to be inspected together with the anatomic information from either a CT or an MRI scan, this software provides several functions for fusing images of different modalities such as PET and CT, PET and MRI, or CT and MRI. In fact, the user can fuse images of any modality. The image fusion is performed by choosing one of seven different co-registration algorithms, which work for intra- as well as inter-modality fusion, regardless of the number of voxels or their sizes. These algorithms include Rigid Motion Correction, Normalised Mutual Information, Normalised Cross Correction, Mutual Information, Entropy Correlation Coefficient, and aligning images either by their centers or to the origin. The main and background images can be freely chosen by the user.

### Implemented Data Analysis and Modelling Methods

Carimas has several implemented methods and algorithms to extract different physiological or pathophysiological parameters from the data. The possible parameters for dynamic data include glucose metabolic rate, myocardial perfusion, receptor binding potential, and oxygen consumption rate. The existing models of Carimas are Patlak [2], Logan [3], fractional uptake ratio (FUR) Index [4], generic compartmental models, and tracer-specific models for several different substances labelled with radioactive isotopes. See “Dynamic PET Imaging” for more details.

### Extensible by Plugin Interface

Because of the Application Programming Interface (API) design of Carimas, different plugins can be first developed and then implemented in Carimas. This enables adding more functions and features to the software so that it fits better to the requirements of a specific research project and the feedback from the professionals can be taken into account without having to create a whole new software version. While some of the plugins have been created by the original Carimas development team, they can be developed by anyone who is familiar enough with the plugin API of Carimas. Using plugins to add new functions to Carimas has a smaller risk of fatal errors compared to creating a new version of the program because, even if there would be some error in the function of the plugin, Carimas only gives an error message for this function instead aborting the whole session and the error is therefore easy to locate and correct. Also, if only the necessary plugins are installed, the actual software requires less computer memory. All of the plugins are free of cost.

### Multiple Image Data Format Support

Currently, most imaging scanners give their output in Digital Imaging and Communications in Medicine (DICOM) format, but many other imaging formats are still in use. Carimas supports several data formats, including Analyze, DICOM, ECAT, Interfile, MicroPET, and NIfTI, and general bitmaps formats such as BMP, JPG, PNG, and TIFF. Carimas can also be connected to Picture Archive and Communications System (PACS) of the hospital.

### Easy to Install and Use

Carimas can be installed on Windows after downloading it from the site https://carimas.fi/carimas-research/. The only system requirement is that the user must have either .NET or Mono framework, both of which are open-source developer platforms by Microsoft and therefore very accessible. The yearly license to the research version of Carimas is 2000 euros and a free trial version is offered. While the research version is for non-clinical use, there also exists Carimas CE, which has CE licensing. The official site of Carimas also provides a user manual and instructional videos can be found from the YouTube channel at https://www.youtube.com/@CarimasTeam.

## Technical Details

### Framework

**Fig. 2:**
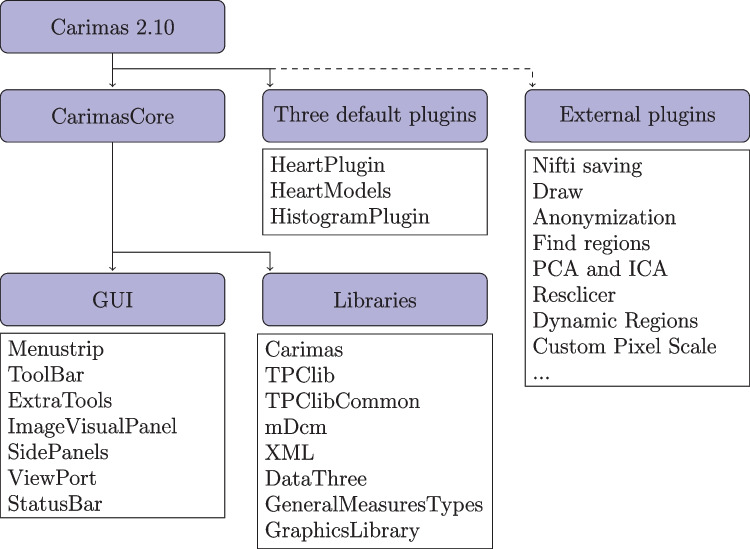
A flowchart explaining the structure of Carimas 2.10

The released package of Carimas (version: 2.10) consists of the core program called CarimasCore and three implemented plugins. CarimasCore has the fundamental functions for data input and output, image visualization, ROI/VOI operations, image fusion, data analysis, modelling, and reporting, as well as the project management and handling of extra plugins. Out of the implemented plugins, HeartPlugin is used for processing cardiac PET imaging data, HeartModels has modelling tools for cardiac imaging, and HistogramPlugin enables drawing histograms from the pixel or the voxel values of the image data. The structure of Carimas is summarized in Fig. 2.

### Graphic User Interface

**Fig. 3:**
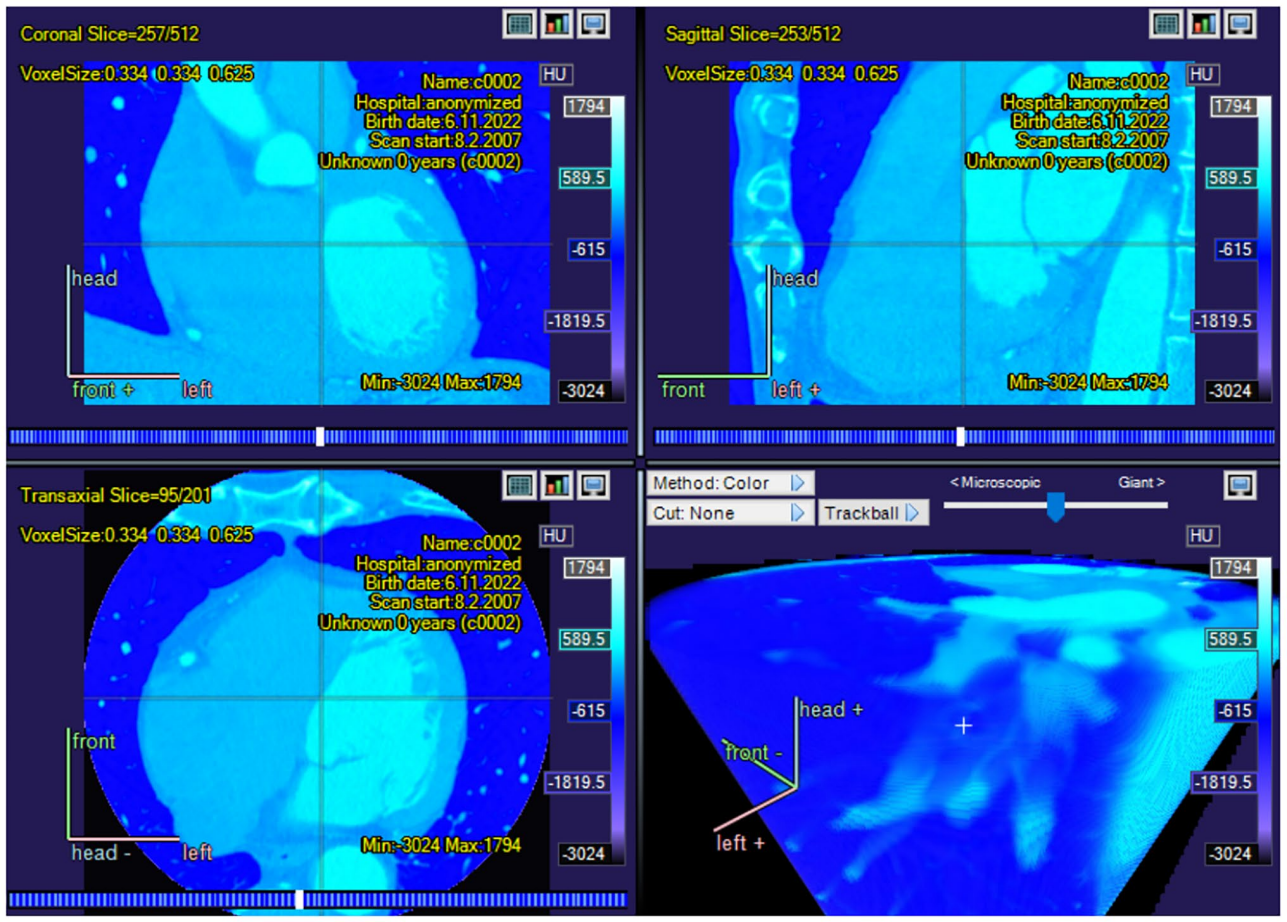
A screenshot showing a CT image of a human heart with color function Ice_edge in Carimas

**Fig. 4:**
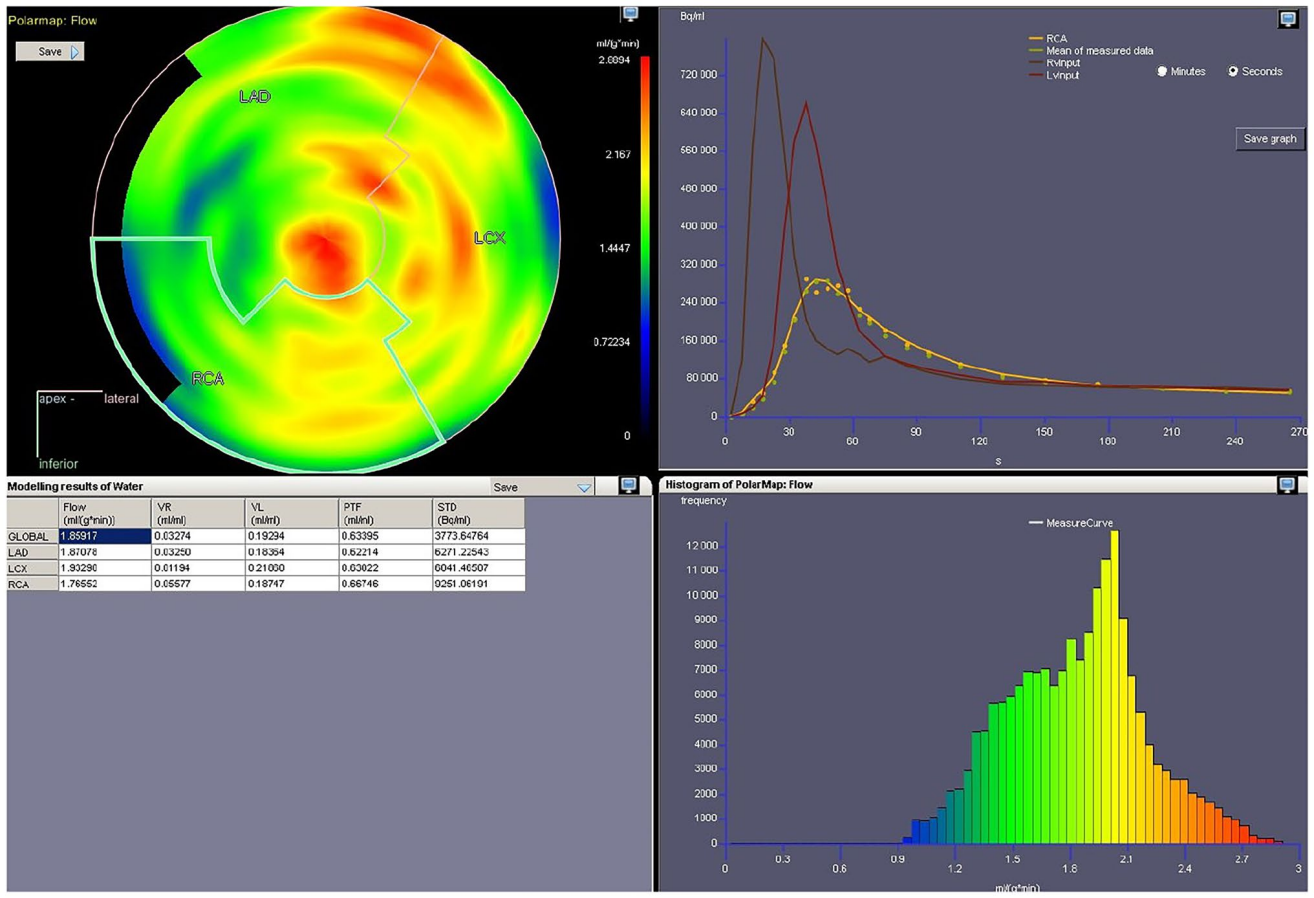
A screenshot of a heart polar map in Carimas showing some of the model functions

**Fig. 5:**
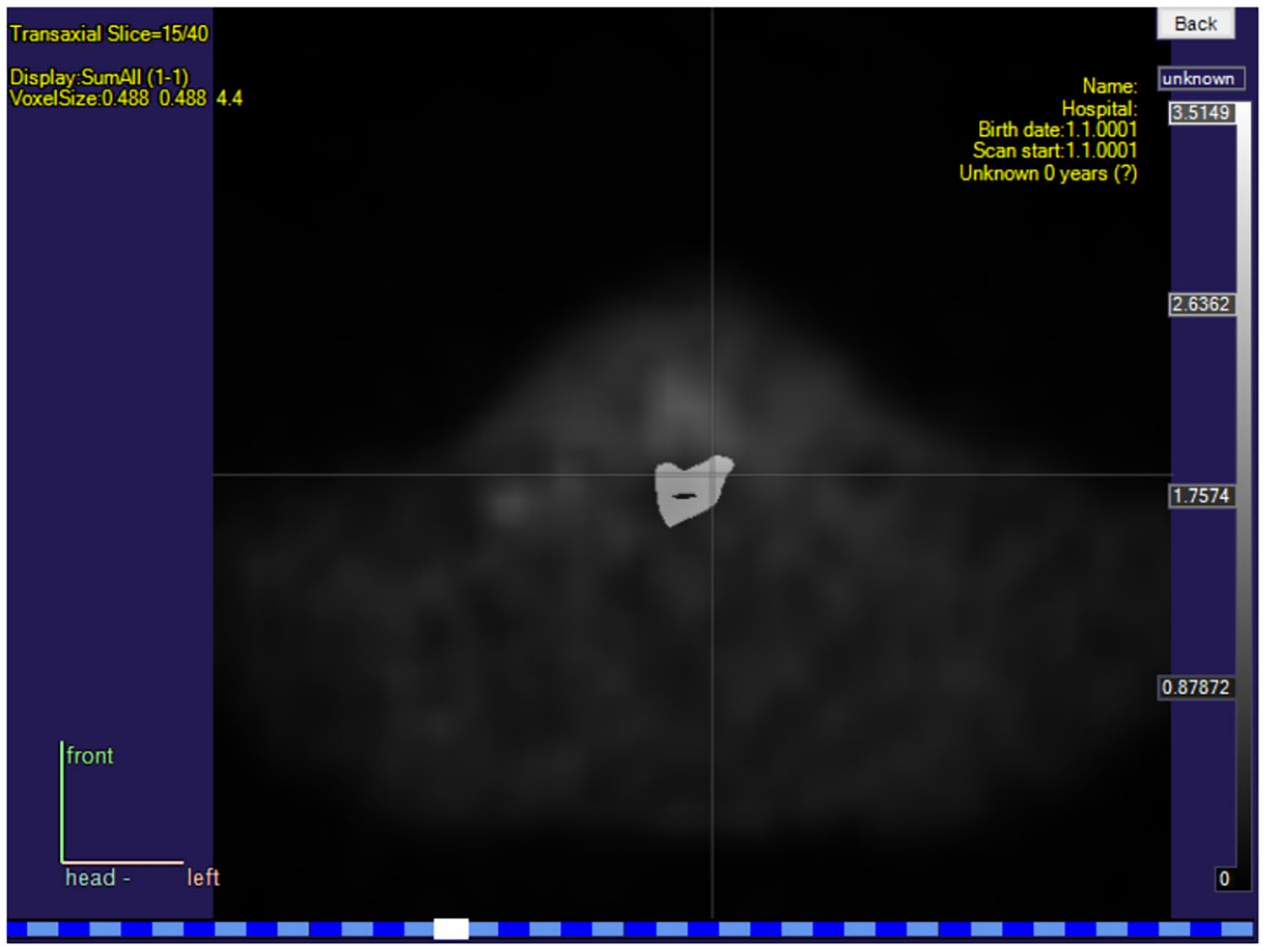
A screenshot showing a ready tumor segmentation mask on a transaxial slice of a PET image of a patient with head and neck squamous cell carcinoma in Carimas

CarimasCore graphic user interface (GUI) consists of seven parts called Menustrip, ToolBar, ExtraTools, ImageVisualPanel, SidePanels, ViewPort, and StatusBar, all of which offer different operations and information. Menustrip has the five typical program menus, including File, Edit, View, Image, and Help, which contain the most fundamental functions in a logical order. For instance, the File menu is mostly used for loading new files and saving the project file. ToolBar has basic tools for zooming and drawing in the images and moving them, while the panels of Carimas window are for controlling the different steps of the workflow and the image view. ViewPort is the visualization window of the imaging data, as shown in Figs. 1, 3, 4 and 5. Additionally, StatusBar displays a progressing status for time-consuming tasks.

### Libraries

The core Carimas has eight different libraries. Out of them, Carimas is the main executable containing main user interface elements and plugin mechanism; TPClib has modelling framework, mathematical functions, image loading, and image formats; TPClibCommon contains common data types; mDcm is has the DICOM format; XML is a simple I/O library for XML; DataTree has the project data structure; GeneralMeasureTypes has certain graphical components used by Carimas displays; and GraphicsLibrary has some low-level bitmap functions. All these libraries are required for Carimas to work, and they also contain the three aforementioned necessary plugins (CarimasCore, HeartPlugin, and HeartModels).

### External Plugins

Currently, around one hundred external plugins have been developed for Carimas and out of them, 27 have been published as dynamic-link library (DLL) files on the Carimas plugin archive at https://turkupetcentre.fi/carimas/files/archive/SecondaryModules4.xml. The most common external plugins for Carimas are related to different file formats. For instance, the plugin called Nifti saving allows the user to save results in the NIfTI file format. Furthermore, plugins contain additional tools for drawing, anonymization of images, generating ROIs more efficiently, and computing more statistics such as principal and independent components from dynamic images. The plugins are downloaded into Carimas with a built-in plugin manager from the Help menu, and the existing plugins can be updated in the same way. The plugins can be coded either in C# or other programming languages as long as a C# wrapper is used. Detailed instructions on on building new plugins will be provided in the plugin archive.

## Examples of Application

### Dynamic PET Imaging

PET is a nuclear imaging technique that measures physiological processes in the living bodies. A radioactive tracer substance is injected or inhaled into the bloodstream of the patient, the substance emits positrons due to its unstable form, these positrons collide with the electrons of the body releasing gamma quanta, and the PET detectors capture the gamma-ray emission from the body [5]. This data from the detectors is reconstructed to create either one static three-dimensional image or a dynamic sequence of several images showing how the tracer spreads into the body as time passes. The dynamic PET imaging can be used to model physiological parameters, such as glucose metabolism, receptor binding potential, and blood flow, that cannot be observed from a static image.

Compartmental modelling means that a physiological system with some dynamic processes is represented by a small number of compartments interacting with each other. These compartments are typically organs, parts of organs, or specific cell or tissue types, and the model explains how blood or other substance flows between them. It is assumed that, upon entering a compartment, the substance of interest instantly mixes with the contents of the whole compartment, and there are no differences in how it is distributed in different parts of this compartment. The speed with which the substance spreads from one compartment to another is either a constant or a simple function and, consequently, the model parameters can be solved from a set of differential equations [6].

In modelling dynamic PET data, generic tissue compartmental models with 1–3 compartments are typically used. For other specific applications, such as $^{15}$O-labeled water cardiac perfusion imaging, a 1-tissue compartmental model is commonly used. Since the researchers tend to generally select a model that is unique for their study, there are several potential options for any given tracer model. For instance, DeGrado et al. [7], Hutchins et al. [8], and Krivokapich et al. [9] have all developed models for $^{13}$N-labeled ammonia cardiac perfusion studies.

Carimas is well-suited for processing dynamic data because this was one of the first requirements for developing the software. Given the multimodality properties of Carimas, the whole dynamic three-dimensional image can be loaded fast and easily into the program, after which it is possible to view it by moving on the time axis. As mentioned already before, the software has several implemented algorithms for computing the needed parameters. Furthermore, Carimas can be used to perform compartment modelling as the heart plugin provides tracer specific models for $^{15}$O-water, $^{18}$F-fluorodeoxyglucose, $^{18}$F-flurpiridaz, $^{11}$C-acetate, $^{82}$Rb-rubinium, and $^{13}$N-ammonia. To apply these models, the user needs to specify the tracer and the desired number of compartments and then choose a model from the resulting list of available options. The model utilizes time-activity curves extracted from the voxels defined by the chosen VOI. Fully customized compartmental models can be also built, after which these new models can be added to Carimas through the plugin structure.

### Heart Analysis

Medical imaging of the human heart is important for treating and diagnosing several heart diseases, including ischemic heart disease. For this purpose, it is often necessary to create a polar map that shows the heart and, in particular, the left ventricular wall. While a medical image of the body is typically a three-dimensional matrix and therefore is shaped like a cuboid, the heart images are often cylinder-shaped as in Fig. 3, the heart itself has a complex shape, and there are differences in its position. Because of this, the optimal two-dimensional picture cannot be found by using a single cross-section of the original three-dimensional image, and one must consider some suitable projection instead [10].

Traditionally, the shape of the left ventricle has been simplified with a geometrical structure shaped like a right circular cylinder with a semi-sphere as an apex [11]. We can present the ventricular walls as a polar map by combining the two-dimensional projections of the spherical surface of the semi-sphere and the curved surface of the cylinder. However, this combination of two coordinate systems is not continuous, which is why another system based on the prolate spheroidal coordinates has also been used for describing the geometry of the left ventricle [12]. This means creating the two-dimensional projection of a surface of revolution for an elliptic curve.

Carimas has a unique workflow for heart analysis. The first step is axis definition, and the other steps of segmentation, analysis, and reporting can be performed after the axes have been specified. The user specifies a long and a short axis so that the ventricular walls of the heart are reoriented and visualized as standard views for cardiac analysis. Using these orientations, Carimas will identify and segment myocardial walls of the left ventricle as well as cavity of the ventricle for the image-derived input function, and then Carimas automatically computes numerical values and creates a polar map as a projection of a surface of revolution for the chosen walls. This type of technique is proven to be reliable for detecting heart defects [13]. Figure 4 shows an example of a ready polar map with modelling tools.

### Tumor Segmentation

After imaging cancer patients, one of the most common routine tasks is tumor segmentation, which means creating a three-dimensional binary mask that shows if a specific pixel in the medical image contains cancerous tissue or not. One tumor mask is presented together with a PET image in Fig. 5. Since segmentation by hand is very time-consuming, Carimas offers several semi-automatic methods. ROIs can be drawn on as rectangles, circles, and ellipses, or as a polygonal, spline, or freehand curve, or by using an automatic contour tool after clicking the starting point. A VOI can be generated by compiling ROIs from several image planes or by using a three-dimensional primitive shape such as a ball or a cylinder. Alternatively, a VOI can be created with a mesh tool that uses interpolation to fill the missing information between the given ROIs. The results of all the segmentation tools are immediately visible and, if they require adjustments, the user can switch to the manual segmentation mode to correct them. Further instructions can be found in the video in the supplementary material of this article. Naturally, these segmentation tools can also be applied for purposes other than for denoting tumors. One of the key advantages of Carimas when compared to other software tools performing segmentation is that segmentation can be performed by using dynamic data. Furthermore, a fully automatic tumor segmentation based on convolutional neural networks will be available in Carimas as a plugin in the summer of 2023.

## Discussion

There are several other software tools similar to Carimas available. The free alternatives are ITK-SNAP, 3D-slicer, ImageJ (Image processing and analysis in Java), Vinci (Volume imaging in Neurological Research, Co-registration and ROIs included), MITK (The Medical Imaging Interaction Toolkit), and Horos, while the commercial options include Corrdor 4DM (Philips), ImageQ (Cardiovascular imaging Technologies), PMOD (PMOD Technologies), and syngo MBF (Siemens). It should be noted that some of the free software tools are not designed for dynamic data and most of the commercial alternatives cannot be extended by the user with plugin interface, though.

What differentiates Carimas from other free and commercial software is the extensive collection of tools for PET quantitative modelling, visualization of 4D PET data, and potential to be extended for additional research and clinical applications. The arguably most unique aspects of Carimas are its compartmental modelling features. It has several functions related to different tracer substances and compartmental models, while many other software tools are primarily designed for static MRI and CT images. Another specialty of Carimas is the wide range of different options related to studying the human heart and, especially, analysis of myocardial perfusion PET. With almost 20 years of lifetime, Carimas has been shown to be well-suited for use in research and clinical analysis. Recently, also a CE-marked version has been released, a significant step in the life cycle of this analysis software.

In the future, the development of Carimas continues in particular for processing dynamic data. Given a new total-body PET scanner arrived in Turku PET Centre in spring 2022, Carimas needs to be used processing very large-sized dynamic PET images, which requires improving the current options for great amounts of four-dimensional data. This also means that the impact of diseases that affect the whole body can be studied better, and new functions for analyzing data of patients with diabetes, coronary artery disease, metastasized cancer, and multiple sclerosis can be implemented to Carimas or its plugins. There is also a plan to develop several plugins applying machine learning for image segmentation and other purposes.

## Conclusion

Carimas is an extendable software that offers numerous different options for visualization, processing, and analyzing medical image data and, unlike some alternative programs, it is easy to use, works well for three- and four-dimensional data, and has several ready models and other functions for dynamic PET data and heart images.

## Supplementary Information

Below is the link to the electronic supplementary material.

Supplementary file1 (ZIP 62 MB)

## Data Availability

Not applicable, no new data was generated.
